# Rapid Release of Doxorubicin from Thermosensitive Liposomes—Contributions of Leakage Versus Unloading

**DOI:** 10.1021/acs.jpcb.5c01564

**Published:** 2025-07-10

**Authors:** Henriette Hummler, Maximilian Regenold, Christine Allen, Heiko Heerklotz

**Affiliations:** † Institute of Pharmaceutical Sciences, 9174 University of Freiburg, Freiburg D-97104, Germany; ‡ Leslie Dan Faculty of Pharmacy, 7938 University of Toronto, Toronto, Ontario M5S 3M2, Canada

## Abstract

Drug release from liposomes loaded by remote loading can proceed via two principal routes: (i) the leakage of the entrapped drug through membrane pores; (ii) the permeation of the drug through the intact membrane as the gradient used for remote loading is collapsed (“unloading”). We assess the contributions of the two release mechanisms for doxorubicin loaded via a pH-gradient into lysolipid-containing thermosensitive liposomes. To this end, release into buffer at physiological pH is compared with release into acidic buffer which should eliminate unloading but leave leakage largely unaffected. Above the transition point at ≈41 °C, unloading contributes ∼30% to the overall fast drug release occurring within 30 s. Immediately below the transition, there is still partial release and partial collapse of the pH-gradient but no substantial unloading. This can be explained by a low permeability of gel-phase lipid for (even deprotonated) doxorubicin and insufficient deprotonation at these pH values.

## Graphical abstract

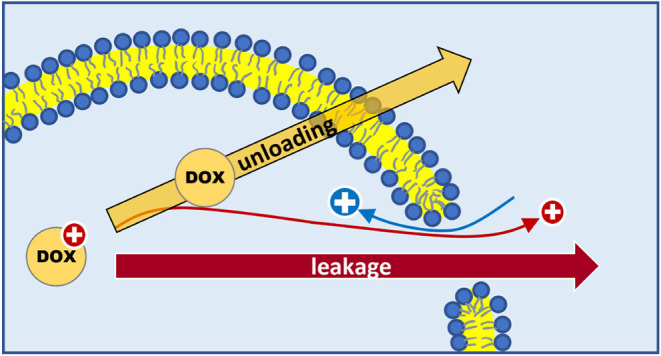

## 1 Introduction

The goal of drug development is to design a dosage form that delivers the drug to the intended site with optimal kinetics, meets all safety and stability requirements and can be manufactured as efficiently as possible. However, these priorities—along with the pressure to accelerate development—can sometimes come at the expense of a deep, mechanistic understanding of the underlying molecular processes. Fundamental biophysical chemistry provides critical insights that can aid in optimizing drug formulations or explaining failures, as in the case of ThermoDox. Such knowledge is essential for developing more effective formulation strategies. This manuscript explores these concepts.

Liposomal formulations represent a drug delivery platform improving the therapeutic efficacy of cancer drugs such as e.g., doxorubicin while limiting their toxicity. The first thermosensitive liposomes developed by Yatvin et al. in 1978 marked an important landmark in the field of nano drug delivery systems.^1^ Later Needham et al. established low-temperature sensitive liposomes (LTSL) containing lysolipid, overcoming difficulties associated with tumor targeting via the enhanced permeability and retention (EPR) effect and limited drug release from traditional liposomes.^2,3^ Applying LTSL in combination with mild hyperthermia (HT) allows for a burst drug release at the melting phase transition temperature (*T*_m_), i.e., at around 41 °C.

LTSL in which doxorubicin (DOX), representing an amphiphatic weak base with a p*K*_a_ of 8.3,^4,5^ is remotely loaded via a pH-gradient, are known under the trade name ThermoDox.^6^ It is the first LTSL formulation that was tested in clinical trials. However, recent setbacks in the OPTIMA trial evaluating ThermoDox in combination with radiofrequency ablation for the treatment of hepatocellular carcinoma do question the targeted application of LTSL [NCT02112656].^7^ Nevertheless, it was concluded that LTSL should not be abandoned. Attention should rather be paid to the right choice of interventional oncology techniques combined with the drug as a multimodal therapy while appropriately planning clinical trials.^7^ Further, the failure of the OPTIMA trial highlights the importance of a thorough mechanistical understanding of the formulation. Even if the significance of *in vitro* tests is often considered limited, they do help predicting more accurately the formulation’s *in vivo* performance.^8^ Furthermore, a new, Thermosome formulation of DOX and other drugs has been developed on the basis of phosphatidyl diglycerol.^9^

The classic, nonthermoresponsive liposomal product Doxil, also known as Caelyx, is a pegylated liposomal formulation into which doxorubicin is remotely loaded via an ammonium sulfate gradient. The release mechanism of this formulation is suggested to be reliant on tumor-specific characteristics.^10,11,15^ As glutaminolysis, leading to the enhanced production of ammonia, is increased in tumor tissue compared to normal tissue, DOX release in tumor tissue is triggered by an influx of ammonia into the liposomes^5,12^ which, in turn, deprotonates doxorubicin. Consequently, the remote loading mechanism is reversed and uncharged doxorubicin is “unloaded” by diffusion through the liposomal membrane.^5^

Thermoresponsive liposomes differ from Doxil in a number of ways. Most importantly, they do not need the circulation times of many hours required for utilizing the EPR effect. Instead, they trigger intravascular drug release upon flowing through a tissue of locally enhanced temperature, relatively soon after injection.^13^ The LTSL formulation uses remote loading of DOX using a pH gradient (with citrate at pH 4 inside). The appearance of membrane pores or defects at 41 °C provides two possible release mechanisms as illustrated in Figure 1, where “unloading” now results from a rise in intraliposomal pH.

**Figure 1:**
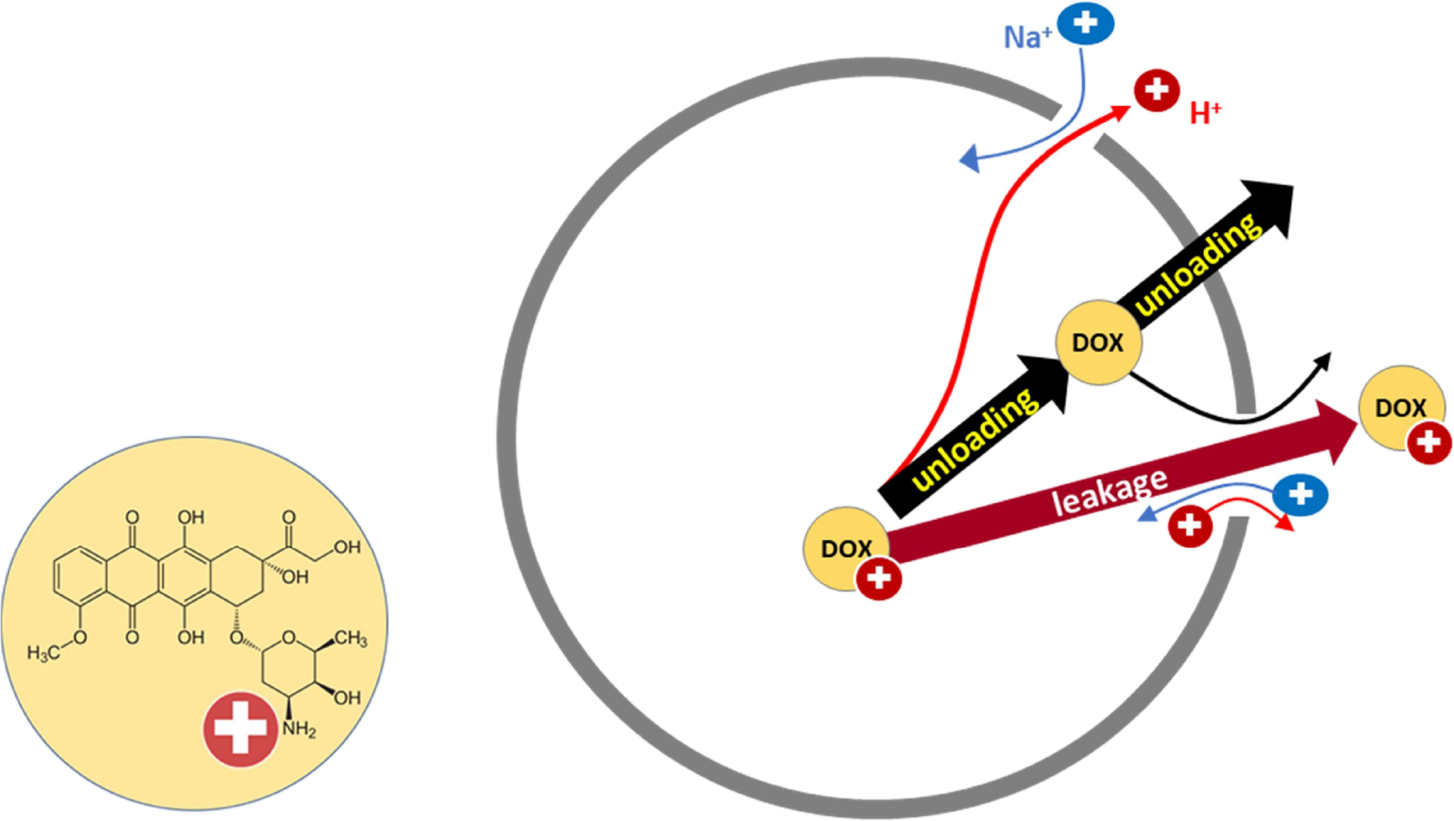
Schematic representation of the two release pathways discussed here. Leakage refers to the efflux of doxorubicin, also in protonated form, through pores or defects in the membrane. Unloading represents the reversal of the remote loading process: proton-cation exchange causes the collapse of the pH gradient. This induces a deprotonation of DOX, which renders it potentially membrane permeant. Experiments at physiological outside pH of 7.4 are assumed to approximate the physiological combination of leakage and unloading—those at outside pH 4 inhibit the pH gradient, DOX deprotonation and, in turn, unloading.

First, DOX could leave the liposome in protonated form via large aqueous defects or pores—this is referred to as “leakage”. Second, “unloading” could be induced as membrane permeabilization for H^+^ and cations such as Na^+^ collapses the pH-gradient. Then, doxorubicin gets deprotonated and diffuses through the membrane in neutral form. Silverman and Barenholz suggested that a similar release mechanism could apply to liposomes encapsulating an amphiphatic weak base remotely loaded by a pH-gradient.^5^ The aim of this paper is to distinguish between these two pathways.

While the mechanism of thermotropic, transient leakage of LTSL has recently been explained in terms of the existence of a small fraction of highly lysolipid-enriched, chain-interdigitated gel domains that melt eutectically with the gel bilayer at the trigger temperature,^8^ the importance of the collapse of the pH-gradient for DOX release has not been addressed yet. This question is not only crucial for a better mechanistic understanding of drug release in general, which seems highly desirable for a more rational optimization of the delivery system. It may also explain individual or tumor-dependent differences of drug release.^4,14–16^

To assess the role of pH-driven DOX unloading from LTSL, we measured DOX release as a function of external pH. In an acidic release medium, unloading effects are inhibited by the lack of a pH-gradient. In contrast, leakage of protonated DOX should essentially be unaffected. Will release be sped up by the possibility of unloading in a nonacidic environment?

## 2 Materials and Methods

### 2.1 Materials

1,2-Dipalmitoyl-*sn*-glycero-3-phosphocholine (DPPC), 1,2-distearoyl-*sn*-glycero-3-phophatidyl ethanol amine-*n*-(methoxy­(polyethylene glycol)-2000) (PEG_2000_-DSPE), and 1-stearoyl-2-hydroxy-*sn*-glycero-3-phosphocholine (lysoPC, in some literature also MSPC) were purchased from Corden Pharma Switzerland LLC (Liestal, Switzerland). Doxorubicin hydrochloride (DOX) was obtained from Tongchuang Pharma Co. Ltd. (Wujiang City, China). Dextran, fluorescein, 40,000 MW, anionic was obtained from Molecular Probes by Life Technologies (Eugene, USA). HEPES, potassium chloride, sodium carbonate, sodium chloride, sodium citrate, and sodium phosphate dibasic were purchased from BioShop Canada Inc. (Burlington, Canada). Dulbecco’s Phosphate Buffered Saline, sodium phosphate monobasic, and Triton-X-100 were obtained from Sigma-Aldrich Inc. (St. Louis, USA). Sodium hydroxide and chloroform were obtained from Caledon Laboratories Ltd. (Georgetown, Canada).

### 2.2 Methods

#### 2.2.1 Preparation of Lipid Films

A protocol by Vigilanti et al. was slightly modified for the preparation of the liposomes.^17^ In brief, DPPC, lysoPC, and DSPE-PEG_2000_ were dissolved in chloroform at a molar ratio of 86/10/4, respectively. The solvent was removed by evaporation. Consequently, the lipid film was dried in a vacuum oven overnight.

##### 2.2.1.1 Preparation of LTSL Loaded with Doxorubicin

Preheated citrate buffer pH 4 (300 mM sodium citrate) was used to hydrate the lipid film over 30 min. One μM lysoPC was added to the citrate buffer to avoid loss of the latter in the liposomal bilayer.^4^ Extrusion was performed at 50 °C three times through double-stacked 200 nm pore-sized polycarbonate membranes (Whatman Inc., Clifton, NJ, USA) at 200 psi nitrogen pressure followed by ten cycles of extrusion through double-stacked 100 nm pore-sized polycarbonate membranes at 400 psi nitrogen pressure. A 10 mL Lipex Extruder from Northern Lipids (Vancouver, BC, Canada) was used. Following, the liposomes were placed on ice for 10 min.

An active DOX loading technique based on a pH-gradient was used. The extraliposomal pH was adjusted to pH 7.4 by adding sodium carbonate buffer (0.5 M Na_2_CO_3_, pH 11). LTSL and DOX solution (5 mg/mL) were mixed to achieve a drug-to-lipid ratio of 0.5 mg:10 mg. Loading was performed at 35 °C for 1 h with constant gentle stirring. Loaded LTSL were placed on ice for 10 min.

Unencapsulated DOX was removed using a Spectra/Por 6 Dialysis Membrane (Spectrum Laboratories Inc., Rancho Dominguez, USA) with a molecular weight cutoff of 50 kDa. The liposomal dispersion was dialyzed against 1 L of HEPES buffered saline for 2 h. Dialysis was continued for at least 12 h after the dialysis buffer was exchanged with fresh HBS 7.4. Afterward, the liposomal dispersion was stored in the fridge at around 5 °C.

To achieve a final lipid concentration of about 50 mM, the liposomal dispersion was concentrated by tangential flow filtration with a MicroKros Hollow Fiber Filter Module (Spectrum Inc., Rancho Dominguez, USA). The tangential filter module was activated using a 20% ethanol solution and it was rinsed with cooled PBS buffer pH 7.4 prior to use.

The obtained LTSL loaded with DOX (DOX-LTSL) were stored at approximately 5 °C and used for experiments during the following 48 h.

##### 2.2.1.2 Preparation of LTSL Loaded with Fluorescein-Labeled Dextran

A passive loading approach was chosen for the preparation of LTSL loaded with fluorescein-labeled dextran (FLD). Hence, the lipid film was hydrated through the addition of a 1.5 mg/mL FLD solution in citrate buffer pH 4 (300 mM sodium citrate) to achieve a lipid concentration of 125 mM. The mixture was kept at 50–60 °C for 30 min with frequent intermittent, gentle vortexing. As for the DOX-LTSL, 1 μM lysoPC was added to the hydration solution.^4^ Following, five freeze thaw cycles were performed. Hydrated lipid film was frozen and kept on dry ice for 6 min before being thawed in a water bath at 50–60 °C for another 10 min. Afterward, the multilamellar large vesicle (MLV) dispersion was put into an ultrasonic bath for 2 min.

Extrusion was performed as described for the DOX-LTSL (Section 2.2.1.1). Removal of the unencapsulated FLD from the LTSL was performed using a CL-4B sepharose gel column. Citrate buffer pH 4 was used to equilibrate the column and to elute the samples. The eluent was collected in fractions of about 1.5 mL. Fractions containing LTSL loaded with FLD (FLD-LTSL) were determined and separated from fractions containing only free FLD by measuring the fluorescence intensity (encapsulation efficiency of around 33%).

Concentration of the liposomes was performed as described for the DOX-LTSL (Section 2.2.1.1). The obtained FLD-LTSL were stored at approximately 5 °C and used for experiments during the following 48 h.

#### 2.2.2 Liposome Characterization

##### 2.2.2.1 Size and PDI

The size and the PDI of the liposomes were measured using a Zetasizer Nano ZS (Malvern Instruments Ltd., Malvern, UK). The samples were diluted 1:100 (V/V) with various buffers (see Table 2 below). One measurement consisted of three replications, each composed of ten runs.

##### 2.2.2.2 Phase Transition Temperature Measurements

All samples were diluted to achieve a lipid concentration of approximately 40 mM. Ten μL of each sample of DOX-LTSL were measured against HEPES buffered saline (HBS) pH 7.4, phosphate buffered saline pH 7.4 (PBS 7.4) and pH 6.5 (PBS 6.5), sodium citrate buffer pH 5.5 or pH 4.0 (SoC 5.5 or SoC 4.0). FLD-LTSL samples were measured against SoC 4.0. A DSC Q100 (TA Instruments, New Castle, USA) was used applying three heating cycles starting from 25 to 60 °C with a heating rate of 1 K/min to each sample. *T*_m_, the temperature of the peak onset (*T*_on_), and the peak width at half peak height (Δ*T*_1/2_) were determined using the TA Universal Analysis Software (TA Instruments, New Castly, USA).

##### 2.2.2.3 Doxorubicin Concentration Measurements

DOX-LTSL were lysed with 10% (w/w) Triton-X-100 and fluorescence intensity was measured with a Cytation 5 imaging reader (BioTek, Winooski, USA). Excitation and emission wavelength were set to 494 and 521 nm, respectively.^4^ HBS pH 7.4 was used for the dilution of the samples as well as to prepare a calibration curve. To consider the influence of Triton on the fluorescence intensity, 10% (w/w) Triton-X-100 was added to each calibration-curve sample.

##### 2.2.2.4 Non-Leakage of Fluorescein-Labeled Dextran Upon Heating

To ensure FLD does not leak out of the LTSL even upon heating, LTSL were diluted in PBS 7.4 and SoC 4.0 followed by heating to 45 °C for 15 min. Following, the samples were kept on ice for 10 min. Both, the two heated samples as well as the sample kept at room temperature were run over a CL-4B Sepharose gel column. Eluent-containing liposomes were collected in 0.5 mL fractions. The fluorescence intensity of each fraction was measured (excitation wavelength: 494 nm, emission wavelength: 521 nm)^18^ using a Cytation 5 imaging reader (BioTek, Winooski, USA). In addition, a sample of the liposomes not yet separated from the unencapsulated fluorescent dye after the extrusion was run over the column.

#### 2.2.3 *In Vitro* Release of Doxorubicin-Loaded LTSL

This assay is based on the fact that DOX fluorescence is self-quenched at the high concentration loaded into LTSL so that the amount of DOX released into a dilute, extraliposomal solution is proportional to the increase in fluorescence intensity. An *in vitro* release assay was performed at 1 K increments from 37 to 45 °C to examine the influence of the pH-gradient between the exterior and interior of DOX-LTSL on the release of DOX. Fluorescence intensity of DOX was measured every five seconds over a five second interval for a total of 10 min using a FluoroMax 3/4 steady-state fluorometer (Horiba Scientific-Jobin Yvon Inc., Edison, USA). Excitation and emission wavelength were set to 494 and 521 nm, respectively.^4^ The cuvette holder was connected to an external water bath, which allowed constant temperature control of the sample. Prior to each measurement, the temperature of 1980 μL of the release medium was measured within the cuvette using an external thermometer. The measurement was started after the desired temperature within the cuvette remained constant for at least 3 min. First, a blank measurement was performed over 40 s, before quickly adding 20 μL of DOX-LTSL stock solution (100 μL DOX-LTSL + 900 μL PBS 7.4) and mixing it quickly with a pipet. The fluorescent measurements were started immediately after the DOX-LTSL were added.

DOX release was measured in phosphate buffered saline pH 7.4 (PBS 7.4, without CaCl_2_ nor MgCl_2_, 280–315 mOsm/kg), in phosphate-buffered saline pH 6.5 (PBS 6.5, KCl 5 mM, SoCl 135 mM, Na_2_HPO_4_ 10 nM, NaH_2_PO_4_ 18 mM), as well as in 300 mM SoC 5.5 and SoC 4.0.

Percentage of released DOX was calculated according to eq 1.

Eq 1 Calculation of the fraction of DOX released.

1 $$\%\;\mathrm{DOX}\;\mathrm{released}=\frac{F\left(t\right)-F\left(\mathrm{never}\;\mathrm{heated}\right)}{F\left(\mathrm{lysed}\right)-F\left(\mathrm{never}\;\mathrm{heated}\right)}$$

where *F*(*t*) is the fluorescence intensity measured at the respective time t, *F*(never heated) is the fluorescence intensity of a DOX-LTSL sample prepared as described above but measured at room temperature, and *F*(lysed) is the fluorescence of a DOX-LTSL sample with the addition of Triton-X-100. For the lysed samples, specifically, 2000 μL of the respective buffer were mixed with 10 μL of a 10% (w/w) Triton-X-100 solution. Twenty μL of the DOX-LTSL stock solution were then added to 1980 μL of this mixture, and incubated for 15 to 30 min prior to a 10 min fluorescence measurement at room temperature, 37, or 45 °C.

#### 2.2.4 Measurement of Intraliposomal pH

A protonation equilibrium of fluorescein controls a sigmoidal variation of fluorescence intensity with pH, with a sensitive range of about pH 5–7.5.^200^

First, calibration lines were established in dilute solutions of FLD (no liposomes) to establish the slope of fluorescence intensity, F, with increasing concentration, i.e., d*F*/d*c*, at various pH values (see Figure S4).

Then, FLD-loaded liposomes of LTSL lipids were injected into PBS 7.4 or SoC 4.0 at various temperatures as described before for DOX-containing LTSL. Excitation and emission wavelength were set to 494 and 521 nm, respectively.^18^

In order to estimate the fluorescence intensity contribution of nonentrapped FLD, *F*_0_, that was not eliminated from the sample upon SEC, we considered the fact that the difference in the fluorescence of the never-heated samples, which have not leaked dye from the liposome interior, results from the change in quantum yield of the outside FLD, which is large at pH 7.4 but negligible at pH 4.0. The signal from the liposome interior should, for never heated samples, not depend on outside pH and hence, cancel out in the difference:

Eq 2: Assessment of background signal *F*_0_ arising from extraliposomal FLD

2 $$\begin{array}{l} F0\;(pH7.4)=F\left(never\;heated,\;pH7.4\right)-F\left(never\;heated,\;pH4.0\right)\\ F0\;(pH4.0)=F0\;(pH7.4)\;x\;[dF/dc]\left(pH4\right)\;/\;[dF/dc]\left(pH7.4\right) \end{array}$$

The data shown in Figure S4 imply that fluorescence at a given concentration is by a factor of [d*F*/d*c*]­(pH 4)/[d*F*/d*c*]­(pH 7.4) ≈ 1/28 weaker at pH 4 than at pH 7.4.^200^

#### 2.2.5 Statistics

The parameters used for the characterization of the liposomes were statistically analyzed by one-way ANOVA combined with a Bonferroni multiple comparison test. The significance level of differences is always stated in terms of the p-value for the individual results. Statistical analysis was performed using GraphPad (Prism 10.1.1 GraphPad Software Inc., La Jolla, USA).

## 3 Results

### 3.1 Phase Transition Temperature Measurements

The “melting” transitions of LTSL in the different dilution buffers started at 40.0 ± 0.2 °C and showed a full width at half maximal heat capacity of 0.55–0.75 K (Table 1 and Figure S1 shows sample DSC curves). Small differences between the values are considered irrelevant for the release behavior studied here.

**Table 1:** DSC Measurements of LTSL Empty and Loaded with Doxurubicin

Sample type and buffer	*n*	T_on_ [°C]	Δ*T*_1/2_ [°C]	*T*_m_ [°C]	enthalpy [cal/g]
empty, HBS 7.4	6	40.2 ± 0.2	0.71 ± 0.06	40.8 ± 0.3	0.19 ± 0.09
loaded, PBS 7.4	7	39.8 ± 0.2	0.68 ± 0.05	40.3 ± 0.1	0.19 ± 0.05
loaded, PBS 6.5	4	39.9 ± 0.1	0.63 ± 0.02	40.4 ± 0.1	0.15 ± 0.02
loaded, SoC 5.5	4	40.2 ± 0.2	0.55 ± 0.04	40.7 ± 0.2	0.14 ± 0.02
loaded, SoC 4.0	4	39.9 ± 0.1	0.75 ± 0.05	40.6 ± 0.1	0.14 ± 0.02

FLD-LTSL show *T*_on_ of 40.5 °C, Δ*T*_1/2_ of 0.9 °C, and *T*_m_ of 41.4 °C.

### 3.2 Characterization of Liposome Size and DOX Concentration

All DOX-LTSL batches were characterized by dynamic light scattering to share *z*-average hydrodynamic diameters within 108–124 nm and polydispersity indices of 0.06–0.09 (Table 2). The minor differences are considered without relevance to the study. For the FLD-LTSL the *z*-average was 119 nm and PDI 0.04.

**Table 2:** *z*-Average and PDI of LTSL Loaded with Doxurubicin

Medium of dilution	n	z-average [nm] (mean and SD)	PDI (mean and SD)
HBS 7.4	6	109 ± 2	0.08 ± 0.01
PBS 7.4	3	108 ± 4	0.09 ± 0.01
PBS 6.5	3	109 ± 5	0.07 ± 0.00
NaC 5.5	3	124 ± 4	0.07 ± 0.01
NaC 4.0	3	116 ± 4	0.06 ± 0.01

The measured average DOX concentration of 2.54 ± 0.11 mg/mL demonstrated the reproducibility of the manufacturing process, ensuring consistent DOX concentrations for the release experiments.

### 3.3 In Vitro Release of Doxorubicin from LTSL

The influence of different extraliposomal pH values, i.e., 4.0, 5.5, 6.5, and 7.4, on DOX release kinetics from LTSL was tested at temperatures between 37 and 45 °C (Figure 2). At 37 and 38 °C, release is very weak and proceeds on a time scale of hours, independently of the release medium.

**Figure 2:**
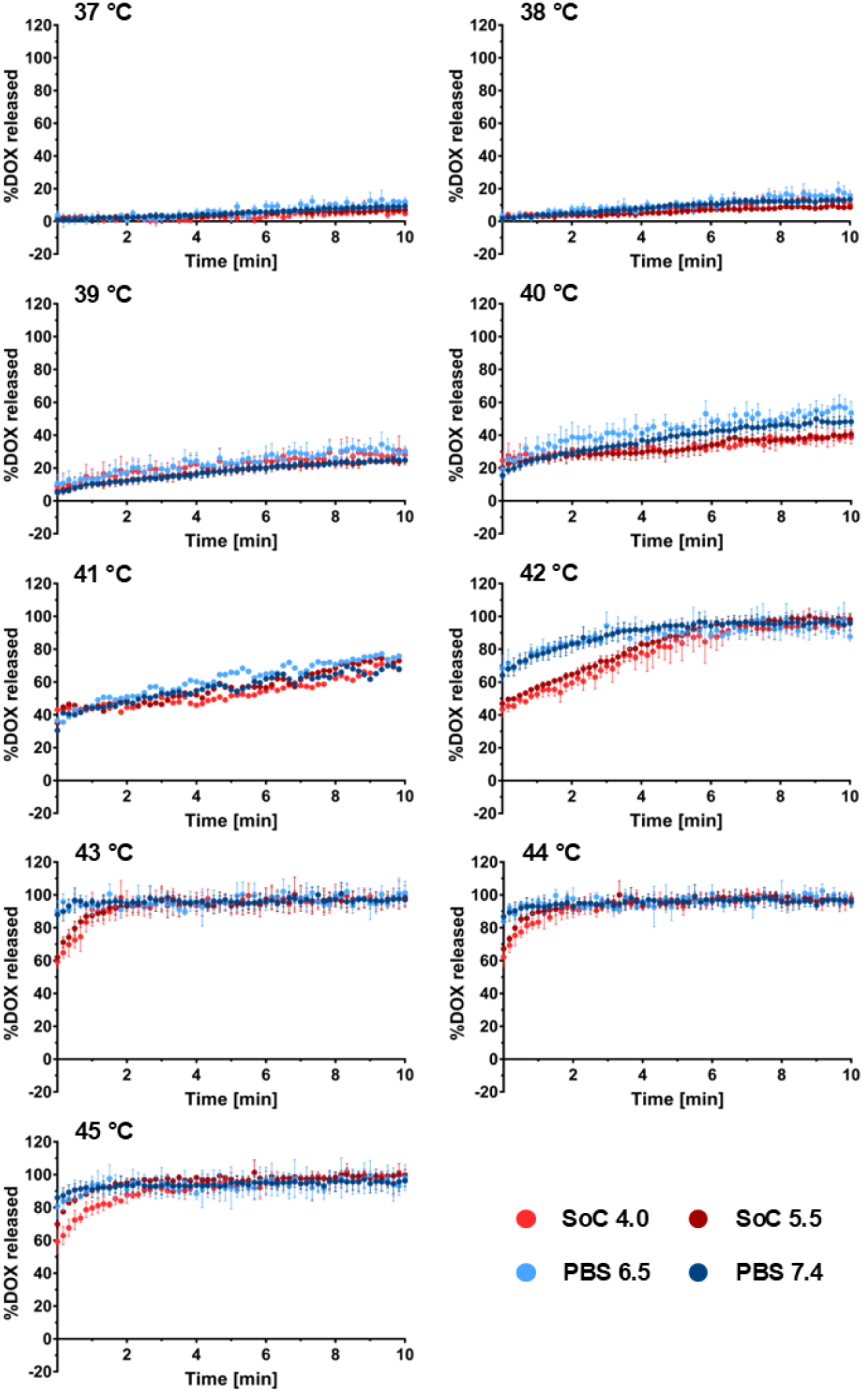
DOX release from LTSL in different buffers at 37–45 °C, unstirred. Standard cuvette assay based on the fluorescent properties of DOX was performed in buffers with varying pH to determine drug release kinetics. Experiments were conducted in triplicates (excluding 41 °C). Data is shown as mean ± SD (*n* = 3).

At and above 39 °C, part of the drug is released very quickly, essentially within the dead time of the measurement of 10 s. The remaining DOX is released much slower. The fraction of fast-released DOX and the rate of slow-release increase with increasing temperature.

A clear and systematic effect of the pH of the release medium is found only at temperatures above 41 °C. In particular, release into an acidic medium shows a smaller fraction of rapidly released DOX.

The standard cuvette assay studying DOX release from LTSL was further performed under stirred conditions (Supporting Information, Figure S2). Through the turbulence created by stirring, the data contain more noise compared to measurements for which no stirring was applied. However, no systematic differences can be found between DOX-release under stirred or unstirred conditions.

### 3.4 Non-Leakage of Fluorescein-Labeled Dextran Upon Heating

To confirm a sufficient and stable entrapment of FLD within the LTSL even at elevated temperatures, we challenged the FLD-LTSL with different stress situations, i.e., temperatures >45 °C in different buffers. Gel filtration gave rise to two fluorescence peaks—free and entrapped FLD—only for the nonpurified, unseparated samples (Figure 3; *unseparated*). All other samples only show one fluorescence peak representing entrapped dye. The obvious variability in peak height and AUC is due to differences in sample concentrations/volumes. However, it does not affect the qualitative analysis of the experiment.

**Figure 3:**
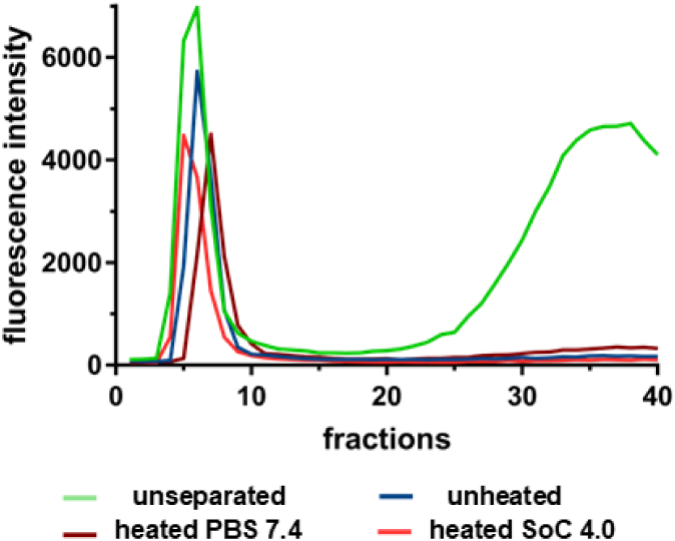
Proof of nonleakage of FLD upon heating. To confirm that FLD is not leaking from LTSL upon heating, unseparated, unheated, and heated PBS 7.4/SoC 4.0 samples were run on a CL-4B Sepharose gel column (*n* = 1). Fluorescence intensity of each 0.5 mL fraction was measured.

### 3.5 Intraliposomal pH Tracking of LTSL in Different Buffers

To better understand the pH collapse during the drug release process, we tracked changes in the intraliposomal pH while heating the LTSL. The fluorescence intensity of FLD depends (at least up to 10 μg/mL) linearly on concentration and, at a given concentration, sigmoidally on pH with a sensitive range at about 5–7 (Figure S4B).

Twenty-μL aliquots of FLD-loaded liposomes prepared with an inside pH of 4.0 were injected into 1.98 mL of SoC 4.0 (Figure 4B) or PBS 7.4 (Figure 4A) buffers at different temperatures and the fluorescence intensity was measured as a function of time after injection. The effective FLD concentration in these experiments is not explicitly known but should, within error, be the same in all samples. These experimental intensity readings were corrected for fluorescence arising from nonentrapped FLD estimated using eq 2 with *F*_0_ values of 6.4 au at pH 7.4 and 2.9 au at pH 4.0. The results were plotted in Figure 4A,B.

**Figure 4:**
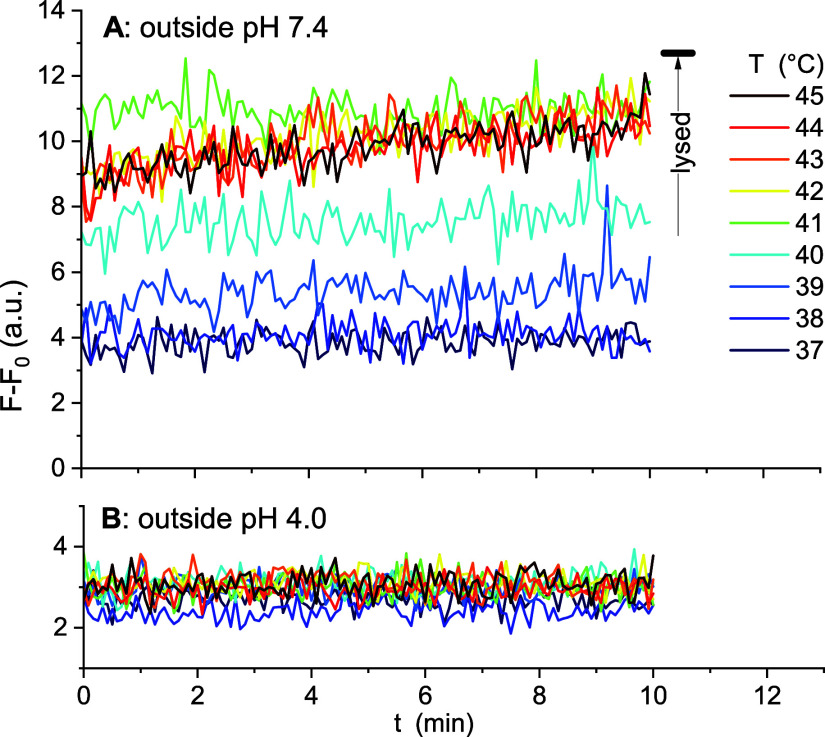
Tracking of intraliposomal pH in different buffers in terms of the pH-dependent, background-corrected intraliposomal FLD fluorescence intensity, *F*–*F*_0_. FLD was loaded into liposomes of the LTSL lipid composition at pH 4.0. Panels A and B show *F*–*F*_0_ recorded as a function of time after injecting aliquots of these liposomal dispersions into PBS 7.4 (panel A) or SoC 4.0 (panel B) of various temperatures—see color coding in Panel A. The black horizontal bar in (A) indicates the average *F*–*F*_0_ after lysing the samples, i.e., after bringing the pH to 7.4.

Injecting liposomes with internal pH of 4.0 into an outside pH of 4.0 (Figure 4B) should leave the pH unchanged, independently of leakage. In line with this, the fluorescence intensities are constant at ≈ 3 ± 1 au, idenpendently of temperature and temperature-induced leakage. Hence, this intensity can tentatively be assigned to pH 4.0. Complete lysis of the liposomes in the large excess volume of pH 7.4 yielded an average value of *F*–*F*_0_ ≈ 12.7 au—this can be assigned to the fluorescence of the FLD concentration used in the experiments at pH 7.4.

Even without a more quantitative assignment of *F*–*F*_0_ to pH, a number of crucial conclusions can be drawn from Figure 4. Up to 38 °C, there is no significant change suggesting the pH gradient between inside 4.0 and outside 7.4 essentially maintained. This also excludes unloading. At 39–41 °C, there is an increasing extent of burst release of protons (drop of the pH gradient) that occurs within the dead time of the measurement of 10 s. Then, the membranes anneal and the intraliposomal pH remains constant and, at leats up to 40 °C, significantly below 7.4. At 41 °C, fluorescence stays at ≈11 ± 1 au, approaching but apparently not fully reaching the signal assigned to pH 7.4.

Above 41 °C, the burst release appears slightly weaker than at 41 °C but now, the membranes do not fully anneal but samples show a slow yet ongoing further increase in fluorescence over the 10 min recorded.

These findings are in line with the DOX release data obtained here (Figure 2) and the mechanism of DOX release described in the literature as discussed below.

The Supporting Information describes an attempt to quantitatively assign pH to *F*–*F*_0_ but this does not add crucial further information in addition to the important conclusions drawn form a more qualitative interpretation above (Figure S4). It suggests pH values of 5.4–6 reached at 39 °C, 5.7–6.3 at 40 °C, 6.3–7 at 41 °C and 6.0–6.8 within 10 min (and further increasing) for 42–45 °C. Finally, it should be mentioned that the absolute pH values are of minor relevance given that the starting pH in LTSL is 5.5 due to the limited buffer capacity upon remote loading and not 4 as used here. In contrast, the crucial conclusion of fast but limited, “burst” steps in intraliposomal pH that occur, to an increasing extent, upon exposure to 39–41 °C, can be assumed to hold true for DOX-loaded LTSL as well.

## 4 Discussion

### 4.1 Fast But Only Partial DOX Release and pH Matching

Both the release curves compiled in Figure 2 and the pH changes shown in Figure 4 agree in indicating three principal temperature ranges. Below 39 °C, both DOX and proton leakage were very weak. At 39–41 °C, there was a growing amount of burst leakage (completed in ≈10 s dead time of the measurement), followed by an annealing of the membrane. Above 41 °C, the burst leakage is followed by slower, further release of DOX and protons. This behavior can be explained with the release mechanism of LTSL.

The LTSL mixture had been empirically optimized to contain 10 mol % of lysoPC to achieve a sharp trigger point of drug release at 41 °C.^2^ No convincing explanation had been found for the fact that the temperature-induced leakage was limited and release stopped before all drug had leaked out. However, release up to 100% was observed for measurements over a longer time period, i.e., 1 h (Figure S3).

Recently, this trigger point at 41 °C and a lysoPC content of 10 mol % turned out to represent a eutectic point of the DPPC-lysoPC mixture, the major lipids of LTSL.^8^ The eutectic below 41 °C consists of a lysolipid-depleted matrix of gel-phase bilayer containing a small fraction of interdigitated gel domains with an extremely high lysoPC content of >33 mol %. As the system is heated beyond the trigger point, both matrix and lysoPC-domains melt at once, giving rise to a fluid bilayer with transient local regions of very high lysolipid content at the positions where interdigitated domains had been before. These regions were argued to form pores until the lysolipid has distributed homogeneously over the whole membrane and the, then averaged, lysolipid concentration has fallen below the pore-formation threshold.^8^ However, the lysoPC-containing fluid phase at >41 °C shows a generally higher permeability than the gel phase, giving rise to some ongoing DOX release and reduction of the pH gradient after the end of the burst phase. This is in line with the fact that loading of DOX is done at 38 °C^14^ to avoid a slow yet significant loss of the pH gradient in the fluid phase.

The partial reduction of the pH gradient shown in Figure 4 reaching different, apparently stable internal pH values depending on outside pH and temperature can be explained by the same interdigitated-domain mechanism. Generally, a partial change in pH could be the result of charge-balancing ion transport across the membrane, finally resulting in the pH-gradient depletion.

### 4.2 Unloading Does Not Enhance Release Below *T*_m_

Let us, first, consider the release curves into different external buffers below 41 °C, which is the approximate midpoint of the LTSL phase transition, *T*_m_.^2^ They comprise a fast contribution occurring within the experiment’s dead time (40% at 41 °C) followed by a slower phase on the order of minutes. The average release within the first 33 s, which represents primarily the fast component, are replotted as a function of temperature in Figure 5. This time regime can be considered primarily relevant for the intravascular drug release from liposomes flowing through a limited volume of hyperthermal tissue.^19^ Interestingly, despite a substantial release already below midpoint *T*_m_, reaching a 33 s average of about 40% at 41 °C, there is no significant effect of pH to be detected. This implies that in this temperature range, unloading plays essentially no role. This is in line with Needham et al., who described the proton efflux at temperatures below *T*_m_ to be associated with the leakage of DOX from lysolipid-containing LTSL at body temperature within the bloodstream.^4^

**Figure 5:**
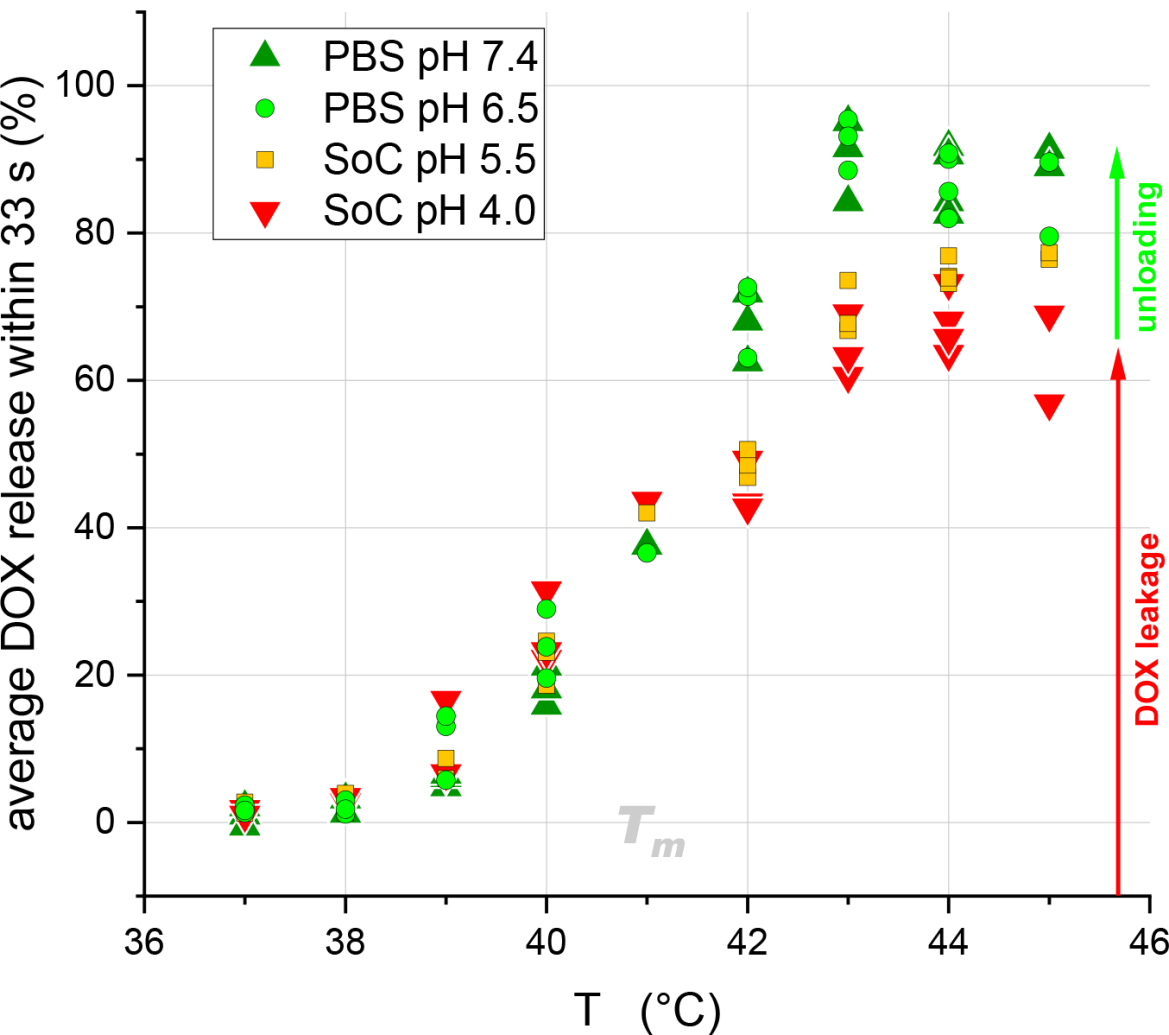
Average percentage of DOX release measured in the first 33 s after exposure of LTSL to buffers of different pH (see legend in plot) at elevated temperatures (see abscissa). Equal symbols at each given T represent measurements of three individual batches of liposomes. The data were obtained by averaging the first 5 data points of the respective data sets presented in Figure 2.

Two effects may contribute to negligible unloading of neutralized DOX through the intact membrane. First, the increase in pH after exposure to 38 and 39 °C may be detectable but still insufficient to produce a substantial fraction of neutralized DOX. This results from the nonlinearity of the Henderson–Hasselbalch equation. For example, for a p*K*_a_ = 8.2, a 1-unit increase in pH from 5.4 to 6.4 would produce 2% deprotonated species while one from 6.4 to 7.4 yields 12%. Second, the permeation of neutralized DOX through the membrane is much slower in the gel phase, present or dominating below 41 °C, than in the fluid phase above. This is also reflected by the standard LTSL loading temperature of 38 °C:^14^ while higher temperatures challenge the pH gradient, lower temperatures slow down loading of neutral DOX markedly.

### 4.3 Unloading Contributes Markedly to DOX Release Above 41 °C

Above the eutectic “melting” point of the LTSL mixture at about 41 °C, fast release is enhanced to outside pH of 6.5 and 7.4 compared to 4 and 5.5 by 20–30 percentage points (≈50%), suggesting that release profits substantially from pH-induced unloading. In this temperature range, the intraliposomal pH approaches the p*K*_a_ and most of the membrane is fluid; this increases the fraction of neutral species and speeds up its unloading by diffusion across the intact fluid membrane.

### 4.4 The Importance of pH-Induced Unloading for the Pharmaceutical Function of the Delivery System

The contribution of unloading to DOX release increases both the amount of quickly released DOX at 42–44 °C and the temperature selectivity as indicated by the slope of the green data points in Figure 5. Both parameters are crucial for optimal function.

Another important question is related to the fact that the increased metabolic activity in tumor tissue tends to decrease the local pH.^5,20,21^ While this is a potential concern for a release involving pH-induced unloading, it may not be problematic for the system at hand. First, the practically full performance in a release medium of pH 6.5 (light green points in Figure 5) suggests that weak changes are not crucial. Furthermore, the strategy of LTSL at this point is to release the drug quickly into the blood vessel—not to have most liposomes extravasate into the tissue before drug release.^13^

To accurately predict the *in vivo* performance of a liposomal formulation, the *in vitro* release assays need to mimic the *in vivo* condition as closely as possible. The release assay conducted here does show some limitations. First, the release media employed in these studies do not precisely mimic *in vivo* conditions in regard to normal serum composition. The different serum components can have crucial influence on the stability and release kinetics of liposomes, e.g., allowing for the formation of protein corona.^22–24^ Second, the average *in vivo* transit time of liposomes through various solid tumors is only a few seconds.^13^ Therefore, good temporal resolution is desired for the first few seconds. The relatively long time scale of the release assay and the absence of data from the first approximately 10 s creates a conflict with this short transit time.^19^ To capture release during the first seconds, another experimental set up would be needed.^22,25^ Third, microfluidic devices may be better to mimic the complex shear stress liposomes are exposed to in tumor microvasculature. Fourth, even though triton is commonly used to lyse liposomes in fluorescence assays, it is not optimal for the fluorescence measurement of lysed samples. The influence of triton on fluorescence intensities has been discussed in the literature.^26,27^ Despite these limitations, the *in vitro* release assay did enable evaluation of the influence of a pH-gradient on the release of DOX from LTSL.

## 5 Conclusions

As previously described, the main mechanism of DOX release from pH-gradient-loaded, thermoresponsive LTSL is based on transient, lysolipid-lined pores occurring upon heating to temperatures around *T*_m_. The partial efflux of DOX through these pores is accompanied by a partial collapse of the pH gradient used for remote loading. This shall deprotonate nonlinearly increasing fractions of DOX as the internal pH approaches the p*K*_a_ of 8.3. At 41 °C, most of the membrane becomes fluid and permits a relatively rapid diffusion of the neutral species across the intact bilayer—a process we refer to as unloading.

Hence, only above the transition temperature, starting at ≈42 °C, the pH-induced unloading contributes substantially to the extent of fast DOX release with about 50% of the release of DOX^+^ through the pores (20–30 percentage points of total fast release). This represents a significant contribution to the thermoresponsive drug release from the liposomes.

The elimination of unloading below ≈41 °C enhances the temperature sensitivity of the LTSL.

The narrow phase transitions and the only partial, burst release of DOX and only partial fast pH matching followed by an annealing of the membrane are in line with a previously described mechanism based on a eutectic behavior of LTSL.^8^

## Supplementary Material



## Notes

Figure S1. Sample DSC curves of DOX-LTSL samples diluted in PBS 7.4, PBS 6.5, SoC 5.5 and SoC 4.0. A DSC Q100 (TA Instruments, New Castle, USA) was used applying three heating cycles starting from 25 to 60 °C with a heating rate of 1 K/min to each sample; Figure S2. DOX release from LTSL in different buffers at 37–45 °C, stirred; standard cuvette assay based on the fluorescent properties of DOX was performed in buffers with varying pH to determine drug release kinetics; experiments were conducted in triplicates; data are shown as mean ± SD (*n* = 3); Figure S3. DOX release from LTSL in different buffers at 41 °C, stirred, over 1 h; standard cuvette assay based on the fluorescent properties of DOX was performed in buffers with varying pH to determine drug release kinetics (*n* = 1); Figure S4. Calibration curves of FLD in different buffers; to confirm the correlation between fluorescence and concentration of FLD (0.3–5.0 μg/mL), calibration curves in media showing different pH values were conducted (*n* = 1) (PDF)
