# Supplementary material for: Rapid Release of Doxorubicin from Thermosensitive Liposomes—Contributions of Leakage Versus Unloading
Source: J Phys Chem B. 2025 Jul 10;129(29):7518–27. doi: 10.1021/acs.jpcb.5c01564 (PMC12302057; doi:10.1021/acs.jpcb.5c01564)
Supplement: Supplementary file 1 [file jp5c01564_si_001.pdf]

electronic supporting information to:

Rapid Release of Doxorubicin from Thermosensitive Liposomes – Contributions of Leakage versus Unloading

Henriette Hummler [1], Maximilian Regenold [2], Christine Allen [2] and Heiko Heerklotz [1, 2,*]

[1] Institute of Pharmaceutical Sciences, University of Freiburg, Freiburg, Germany
[2] Leslie Dan Faculty of Pharmacy, University of Toronto, Toronto, Ontario, Canada
* Correspondence: heiko.heerklotz@pharmazie.uni-freiburg.de

Journal of Physical Chemistry B, 2025

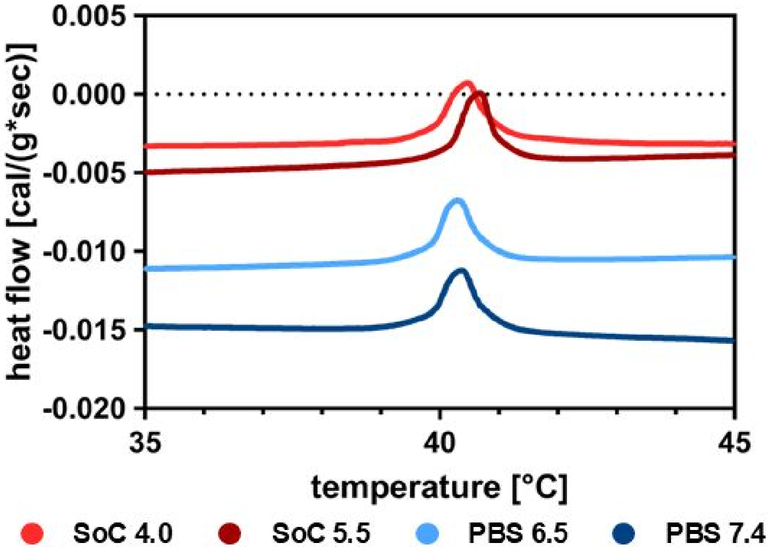

Figure S1. Sample DSC curves of DOX-LTSL samples diluted in PBS 7.4, PBS 6.5, SoC 5.5 and SoC 4.0. A DSC Q100 (TA Instruments, New Castle, USA) was used applying three heating cycles starting from 25 to 60 °C with a heating rate of 1 K/min to each sample.

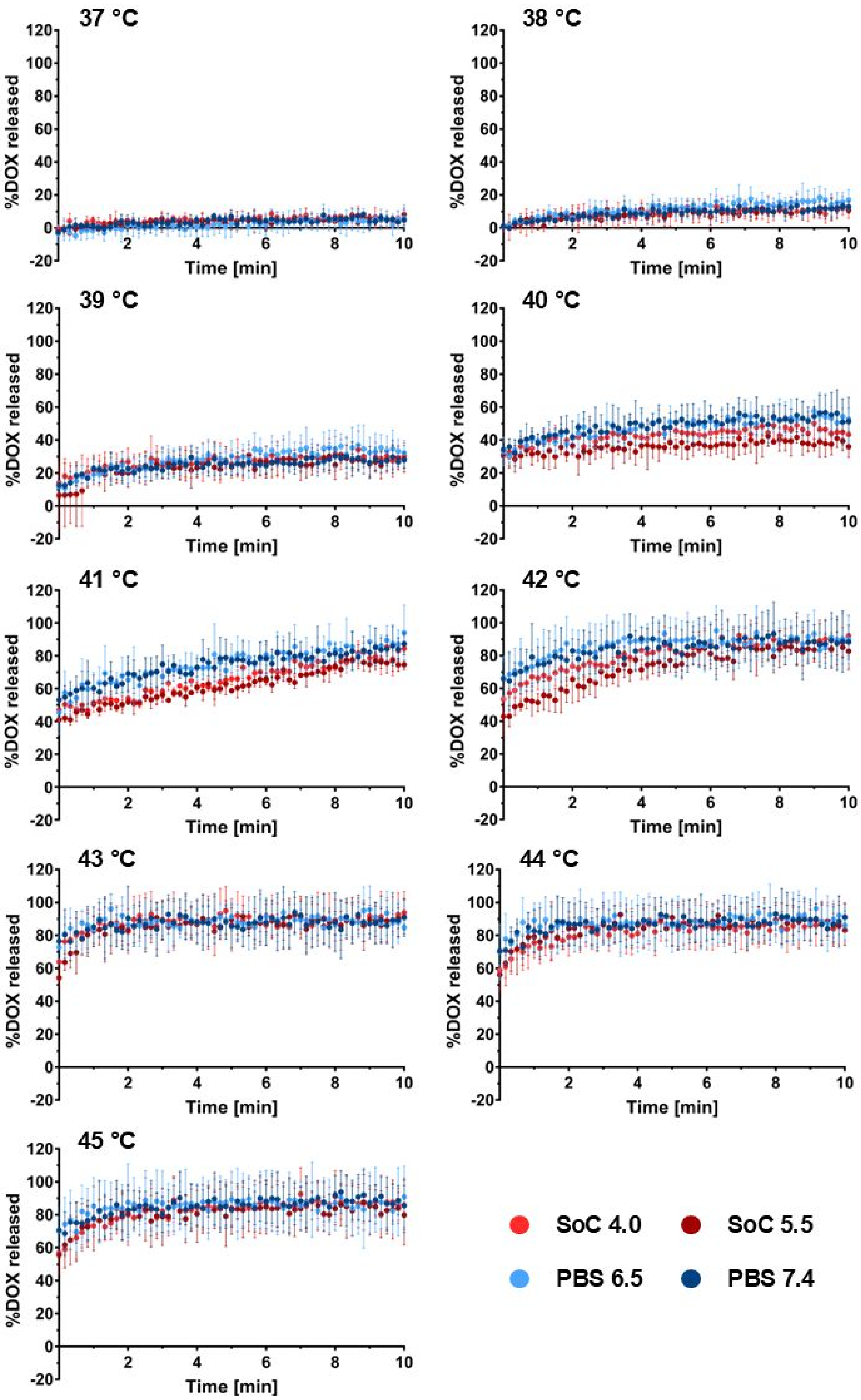

Figure S2. DOX release from LTSL in different buffers at 37 – 45 °C, stirred. Standard cuvette assay based on the fluorescent properties of DOX was performed in buffers with varying pH to determine drug release kinetics. Experiments were conducted in triplicates. Data is shown as mean ± SD (n=3).

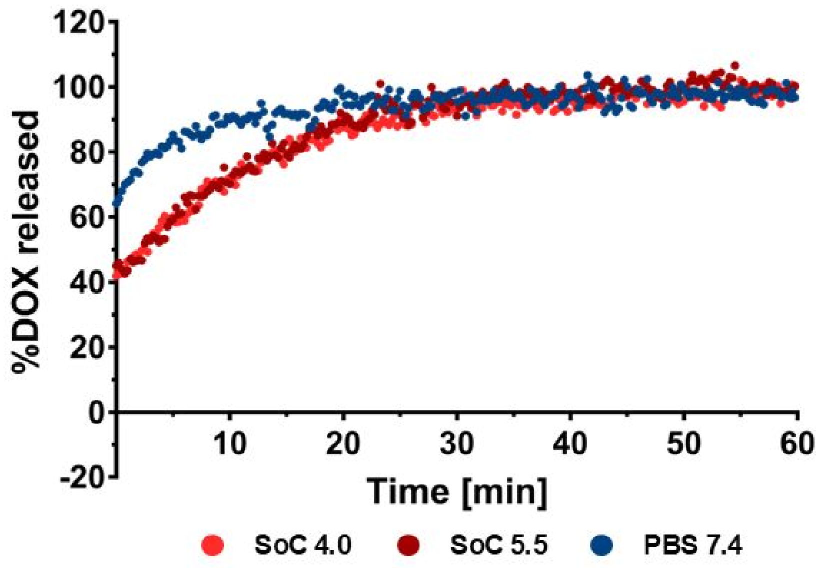

Figure S3. DOX release from LTSL in different buffers at 41 °C, stirred, over one hour. Standard cuvette assay based on the fluorescent properties of DOX was performed in buffers with varying pH to determine drug release kinetics (n=1).

Correlation of intraliposomal FLD fluorescence intensity, F-F_0, with pH

While relative changes in F-F0 can be considered good indications of relative changes in pH in a range between 5 and 7, absolute assignments suffer from a number of critical assumptions and error sources.

Fig. S4A shows linear fits of F measured in FLD solutions (no liposomes, no F0 resulting from extraliposomal FLD) for concentrations up to 5 µg/mL. The slopes of these lines are displayed as a function of pH in Fig. S4B; for pH 7.4, it amounts to 37 a.u./(µg/mL).

With the latter value and the the signal after lysis in PBS 7.4, 12.7 a.u.., we can now estimate the overall FLD concentration present in the assays injecting FLD loaded liposomes into excess buffer (Fig. 4 in main text) as (12.7/37) µg/mL=0.34 µg/mL. The right axis of Fig. S4B now converts the slopes, intensity-per-concentration, to intensities for this concentration of 0.34 µg/mL, reaching 12.7 a.u. at pH 7.4.

The sigmoidal curve now allows for reading pH values for given intensities, but this is not perfectly in line with the ≥ 2 a.u. reading at known pH 4 in Fig. 4B (main text), which is assigned by the grey line in Fig. S4 to a pH >5.3. This independent test of the correlation curve implies that the F-F0 readings might be about 2 a.u. too high. The most straightforward explanation for this deviation would be an underestimation of F0 by ≈ 2 a.u.. Note that this would be a moderate change of F0, assigned to 6.4 a.u. by a fairly crude estimation. While neglecting the residual fluorescence of free FLD at pH 4.0 should not matter (it should only be ≈3.6% of that at pH 7.4 considering the ratio between the dF/dc values), the approach also neglected potential effects of the liposomes (e.g., light scattering) and the high local FLD concentrations within the liposomes.

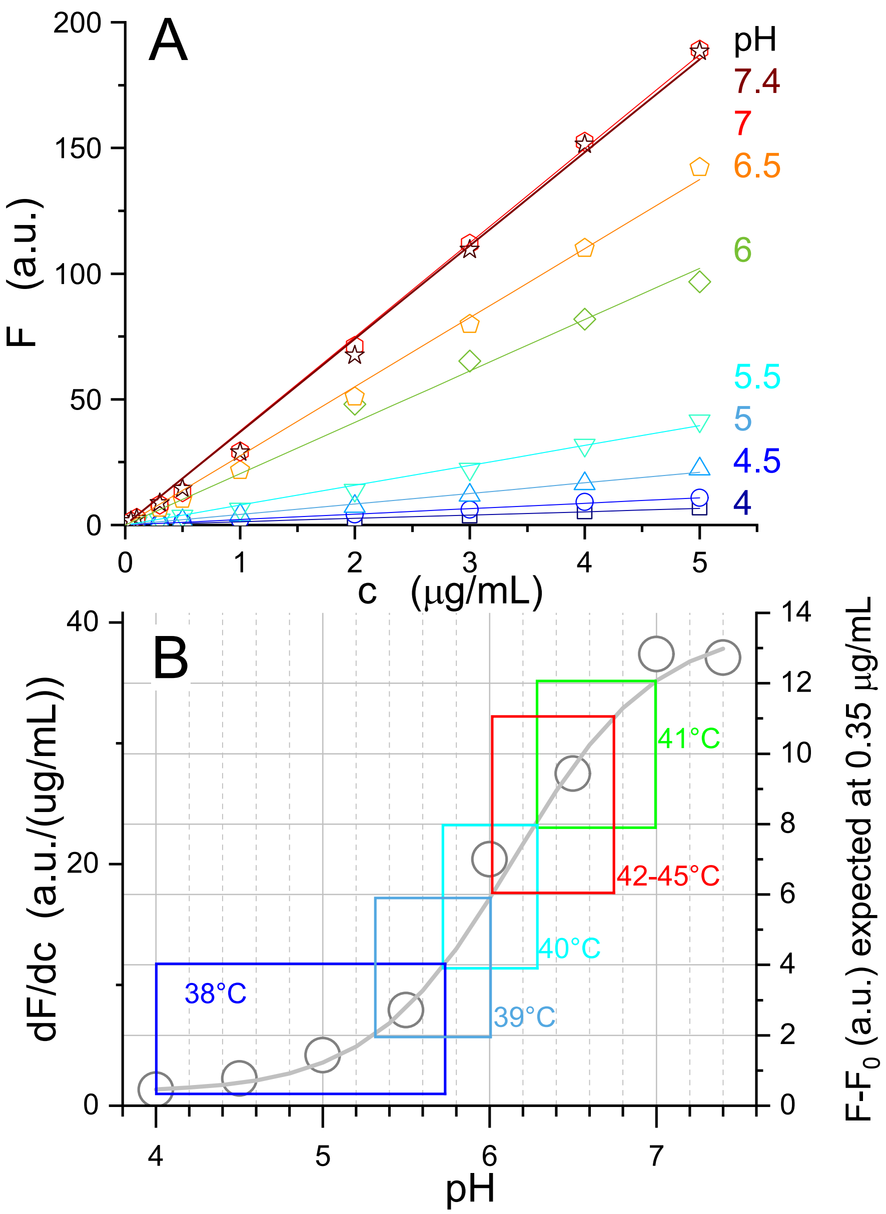

Figure S4. Assessing the relationship between FLD fluorescence intensity, F, and pH. A: Calibration curves of FLD fluorescence intensity, F, in different buffers (pH see plot). B displays the slopes of the lines in panel A (dF/dc) as a function of pH. The right axis shows the corresponding absolute intensities for a concentration of 0.34 mg/mL, which yields the intensity for pH7.4 which has been obtained for $F\text{-}F_0$ after lysis in PBS 7.4 (see Fig. 4, main text). Colored boxes indicate intensity ranges obtained at different temperatures in Fig. 4 and the corresponding expected intraliposomal pH.

For a tentative assignment of pH ranges to the F-F0 data in Fig. 4 (main text) illustrated by the colored boxes in Fig. S4, we have therefore allowed for the possibility of a 2 a.u. overestimation (e.g., including a range of 2—6 rather than 4—6 a.u. for 39°C). This gives rise to the result mentioned in the main text, pH 5.4—6 to be reached at 39°C, 5.7—6.3 at 40°C, 6.3—7 at 41°C and 6.0—6.8 within 10 min (and further increasing) for 42—45°C.
